# XRecon: An Explainbale IoT Reconnaissance Attack Detection System Based on Ensemble Learning

**DOI:** 10.3390/s23115298

**Published:** 2023-06-02

**Authors:** Mohammed M. Alani, Ernesto Damiani

**Affiliations:** 1 Cybersecurity Research Lab, Toronto Metropolitan University, Toronto, ON M5B 2K3, Canada; 2 School of IT Administration and Security, Seneca College, Toronto, ON M2J 2X5, Canada; 3 Center of Cyber-Physical Systems (C2PS), Khalifa University, Abu Dhabi 127788, United Arab Emirates; ernesto.damiani@ku.ac.ae

**Keywords:** IoT, attack, detection, reconnaissance, machine learning, XAI

## Abstract

IoT devices have grown in popularity in recent years. Statistics show that the number of online IoT devices exceeded 35 billion in 2022. This rapid growth in adoption made these devices an obvious target for malicious actors. Attacks such as botnets and malware injection usually start with a phase of reconnaissance to gather information about the target IoT device before exploitation. In this paper, we introduce a machine-learning-based detection system for reconnaissance attacks based on an explainable ensemble model. Our proposed system aims to detect scanning and reconnaissance activity of IoT devices and counter these attacks at an early stage of the attack campaign. The proposed system is designed to be efficient and lightweight to operate in severely resource-constrained environments. When tested, the implementation of the proposed system delivered an accuracy of 99%. Furthermore, the proposed system showed low false positive and false negative rates at 0.6% and 0.05%, respectively, while maintaining high efficiency and low resource consumption.

## 1. Introduction

The Internet of Things (IoT) is made up of a huge number of heterogeneous devices connected to the Internet, including sensors and/or actuators [1]. According to [2], the adoption rates of IoT devices in various application domains are rapidly accelerating. By the end of 2022, the number of connected devices online had exceeded 42 billion. IoT devices tend to have low memory, limited processor speed, and less storage space compared to general-purpose computers. However, because of these limitations, they have low power consumption.

**Figure 1:**
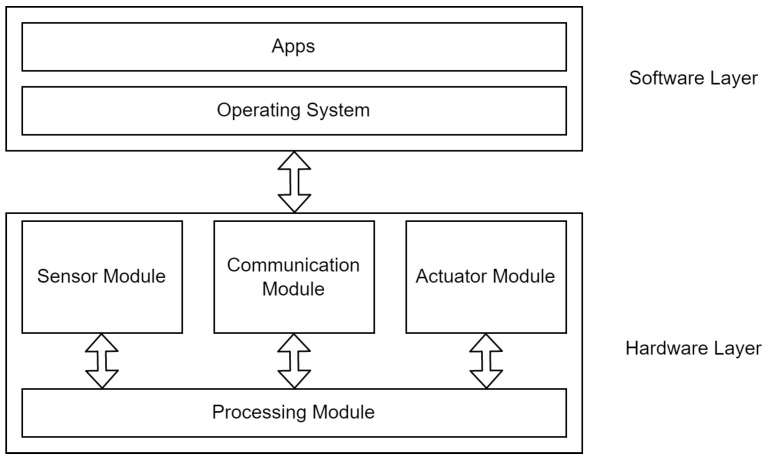
Logical layout of IoT devices [5].

Figure 1 shows some logical components found in most IoT devices. The hardware layer of an IoT device usually includes a low-power microprocessor or a microcontroller along with a memory unit and some type of storage component, such as a secure digital card (SD card), or a flash disk. On top of the processing unit lies a communication module, such as a wired or wireless network interface card, which facilitates Internet connectivity. Most IoT devices also contain an input component of some sort that enables data collection, such as a temperature sensor or a camera.

In most cases, IoT devices also contain some type of actuator. This component of the system is expected to perform a certain task, such as a switch that enables or disables an air conditioning system. Industrial IoT (IIoT) systems, specifically, may include large industrial actuators, such as those that control water treatment facilities or power generation plants.

Above the hardware layer, two software layers can be found: an operating system (OS) and the applications running on top of the OS. While there are many operating systems, only a few are commonly used for IoT devices, including Android [3] and Raspberry Pi OS [4]. The separation between operating systems and applications is not always clear, because most IoT devices are designed to perform a specific task and their applications are tightly coupled with the OS, rather than accessing a standard system call interface. In some devices, the two software layers are combined to create a “firmware” that performs both tasks.

IoT devices can be part of “intelligent” products, such as smart light bulbs or thermostats. They are also common within healthcare and lifestyle support systems such as fitness trackers, smart insulin pumps, smart pacemakers, etc. Other areas of application include unmanned vehicle technology, home appliances, and precision agriculture equipment.

**Figure 2:**
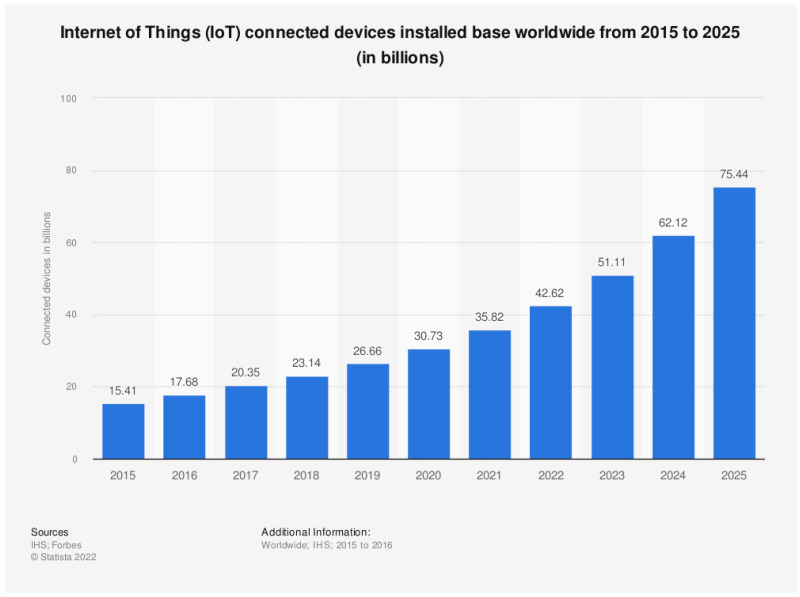
Historical data and expected growth in IoT devices [6].

Ubiquity, combined with low cost and abundant applications, has resulted in widespread adoption of IoT devices in recent years. Available data suggest further growth in connected IoT devices [6], and the number of devices is expected to exceed 75 billion in 2025. Figure 2 shows the historical growth in connected IoT devices, in addition to the projected growth for the next three years.

As shown in the figure, IoT devices have seen steady growth in numbers. While this growth might seem linear in the early years, it has become more exponential as the developments in various areas of application combined with improved user acceptance have fuelled this exponential growth. This growth in the adoption of IoT devices, combined with the lack of security focus by many device manufacturers, has made IoT devices a popular attack target. Vulnerable IoT devices have been hijacked and exploited to carry out large-scale attacks such as Cloudflare [7].

### 1.1. Research Contributions

Applications of artificial intelligence–machine learning (AI–ML) techniques in cybersecurity are designed to help automate and simplify the tasks of detecting, tracking, and blocking intruders. In this paper, we address the challenge of reconnaissance attacks that attackers perform in preparation for large-scale campaigns using an explainable ML-based system. The main contributions of our research work can be summarized in the following points:

- Building an effective, efficient, and accurate ML-based system to detect and counter reconnaissance attacks on IoT devices based on ensemble learning.
- Utilizing recursive feature elimination (RFE) in the process of selection of the most effective features for the detection process in the proposed system. This method provides an advantage in reducing the amount of data collected at the acquisition stage, in addition to reducing the number of features passed to the classifier. In turn, this improves efficiency and reduces the computational burden on IoT devices in the detection process.
- Significantly increasing the trust in the proposed solution by explaining the impact each feature has on the model’s decisions by utilizing SHAP explanations. Such explanation ensures that the decisions made do not originate from a black-box system.
- Produce a preprocessed, balanced, and feature-reduced version of the dataset that includes the most effective seven features only to help facilitate future research.

### 1.2. Paper Layout

The next section of the paper contains some background information on reconnaissance attacks and the corresponding threat model in the IoT context. Section 3 summarizes previous work in this area, with subsections focused, respectively, on ML-based and classical solutions. Section 4 outlines our proposed system. The dataset used in our experiments is presented in Section 5. The following section explains the details of our methodology and experimental setup, including the training and testing strategy and the implementation environment. Section 7 shows the results obtained in each phase of the experiments. Section 8 addresses the explainability of our model, and Section 9 provides a discussion of the results obtained and comparison with previous works. The last section draws our conclusions and outlines some directions for future work.

## 2. Reconnaissance Attacks

Reconnaissance attacks are designed to gather information about potential targets for attacks, such as undefended devices and services [8]. The purpose of reconnaissance is to collect pre-attack intelligence about networks, active hosts within a network, and services running within these hosts. Additionally, reconnaissance attacks help the attacker derive a map of the target network and its IP addressing scheme.

The information collected in reconnaissance attacks is used to identify exploitable vulnerabilities that can be used in future attacks. Reconnaissance is the starting step within the “cyber kill chain” [9], and is usually followed by the weaponization, delivery, and exploitation steps [10].

Reconnaissance attacks employ different techniques to collect information on different aspects of target systems. Some of the most common techniques are listed below [5].

1. Ping Sweep: The use of the Packet INternet Grouper (PING) tool to collect the Internet Protocol (IP) addresses of all active hosts. This type of attack performs pings on all IP addresses within the address range of the network in an attempt to find connected and active hosts.
2. Port Scan: Once the ping scan has detected an active IP address, the attacker will try to find the open TCP ports at that address by sending the first segment of the three-way handshake procedure needed to establish a Transmission Control Protocol (TCP) connection. For the User Datagram Protocol (UDP), scanners send empty user datagrams (UDP) to a range of popularly used ports (within the 1–1000 range). UDP is a connectionless protocol, so it is harder to scan than TCP; usually, there is no answer to pings sent to open UDP ports, but the victim may send an ICMP packet as a response when receiving a ping on a closed UDP port. Alternatively, the attacker can perform a full port scan to find all active ports (within 1–65,536) for both TCP and UDP protocols.
3. Service Enumeration: The attacker tries to gather information about the specific types of service running on the ports found open in the previous step. This is performed by banner grabbing, i.e., by capturing the banner information that is transmitted by the victim’s port when a connection is initiated, to identify application protocols and their specific versions.
4. OS Detection: In this attack, tools such as nmap [11] are used to probe the remote computer. Based on the victim’s responses to valid and invalid TCP packets, nmap can detect the victim’s protocol implementation and infer the operating system it is using. Although such a technique might not give conclusive results on the specific OS version (as several versions of an OS may share the same implementation of TCP), it can help the attacker spot victims running obsolete OS versions where known vulnerabilities can be exploited.
5. Packet Sniffing: In this attack, the attacker does not probe the victims, but passively captures TCP/IP packets that flow through the network. When this traffic is not encrypted, the attacker can sort it by application protocol and address, potentially creating a map of the network and inferring the client or server role of the network node. Although passive packet sniffing can be partly prevented by traffic segregation, at least on wired switched networks, it is still used by eavesdroppers on wireless networks based on traffic broadcast.
6. Open Source Intelligence (OSINT): OSINT is the process of collecting information about a target without directly interacting with the target. This attack takes place by the attacker collecting information about the target from publicly available information such as Domain Name System (DNS) records, domain registrar information, and social media. DNS reconnaissance, in particular, can disclose the network infrastructure without alerting victims. Many organizations do not monitor their DNS traffic, or limit monitoring to zone transfers attempts. Currently, there are online services that provide OSINT for a fee, such as Shodan [12] and Censys [13].

To better understand the dangers of reconnaissance attacks, we will describe a sample scenario that characterizes how these attacks are performed with the steps listed below.

1. The attacker starts by obtaining freely available DNS data on a domain named sample-domain.com from the authoritative DNS server, which is not suspicious.
2. The attacker uses the nmap tool to find the services running on the web server mentioned in the DNS record obtained earlier, using the command nmap -Pn -sSV -A -p- -T5 target-ip-address.
3. With the response of the previous step, the attacker sends a Hypertext Transfer Protocol (HTTP) request to the server, which responds with an http-title message of “Welcome to the Drupal Site”. This tells the attacker that the targeted web server is hosting a website using the Drupal Content Management System (CMS) [14].
4. The attacker decides to use “Droopscan”, a reconnaissance tool designed to collect information about websites that use Drupal CMS.
5. Using the tool, the attacker learns that the version of Drupal CMS used by the victim is within the range of 7.22 to 7.26.
6. The attacker searches Mitre’s CVE [15] database, a public data source that contains information on known vulnerabilities in many systems, and finds a vulnerability called “Drupageddon” [16], also known as CVE-2014-3704, which exists in Drupal CMS versions below 7.32. This dangerous vulnerability, when exploited, can give the attacker remote shell access to the hosting server.
7. The attacker searches the world’s most widely used (and free) exploitation-framework, Metasploit [17], for working exploits, and finds one.
8. The attacker activates the exploit and uses it to gain remote shell access.
9. From there, the attacker can collect usernames and passwords, perform privilege escalation, etc.

It is clear from the above steps that reconnaissance attacks, if not stopped at an early stage, can cause a lot of damage. In the above scenario, the first five steps are classified as reconnaissance activities. If these steps can be detected and stopped, for example, preventing connection requests made using the nmap tool, the attack would have failed despite the existing vulnerability.

### 2.1. Threat Model in IoT Context

An interesting report was published in [18]. The findings of the report show how vulnerable IoT devices are in the real world. The report mentions that 98% of all IoT traffic is not encrypted. Furthermore, 57% of the devices are vulnerable to medium- or high-severity attacks. Although most of the vulnerabilities that can be exploited to conduct these attacks can be easily patched, many IoT vendors do not care to distribute patches, even to well-known vulnerabilities. Furthermore, many IoT devices suffer from design or deployment flaws that make them insecure [7]. The report also mentions that the most significant threat to IoT devices is botnet malware. A botnet is a collection of infected devices managed by a malicious actor, named the bot herder, to perform different tasks such as spreading malware or attacking other targets [19]. Infected devices are always on the hunt for new vulnerable devices to infect and add to the botnet.

Mirai botnet was one of the most renown botnets due to the magnitude of the attacks carried out by it [20]. Since its creation in 2016, it has hit the headlines of cybersecurity news with multiple massive orchestrated distributed denial of service (DDoS) attacks. The first noticeable Mirai attack occurred in September 2016, and it was able to deny access to major online service providers such as Dyn, OVH, and Krebs on Security. The magnitude of this attack reached a rate of more than 1 Tbps, exceeding all previous DDoS attacks at that time. The attack was carried out using a large number of IoT devices, such as surveillance cameras and small home routers. According to [21], there was a time when the Mirai botnet was in control of more than 600,000 vulnerable internet-connected IoT devices.

Mirai’s source code was released by its creator on the Dark Web in 2017. This release spawned multiple variants of Mirai that were based on its source code. These variants were used in many large-scale attacks, such as Katana and Mukashi [22,23,24].

Cloudflare, a well-known cloud service provider, was hit with a large-scale DDoS attack in September 2020 [7]. While the attack only lasted for less than three minutes, it registered a surprising incoming traffic rate of 654 Gbps. Later, Cloudflare identified the attack vector as Mootbot, a Mirai variant. This botnet used 18,705 devices hosted in more than 100 different countries to carry out the attack. Just as in the case of the original Mirai, the botnet exploited well-known vulnerabilities that had not been patched for a long time.

In typical botnet behaviour, after a botnet malware establishes contact with command and control (C&C), it starts scanning for other IoT devices within the local network, and in neighbouring networks [25]. Mirai variants start by scanning for active hosts on the network and then move on to deeper scanning to find which active hosts on the network are actually IoT devices. Once they find an IoT device, Mirai variants start performing deeper scanning to find out the victim’s specific type and model based on a set of signatures. Once the device is identified, the botnet starts to exploit default credentials to gain access to it, infect it, and start the cycle all over again. This behaviour shows that reconnaissance attacks play a significant role in the spread of malware in general and of botnets in specific. The main reasons why Mirai and other botnets were successful in spreading can be summarized as follows.

- Most commercial IoT devices do not receive regular patches from manufacturers, as security is not a priority concern for these manufacturers.
- Even when manufacturers issue patches to discovered vulnerabilities, the patching process is often complicated and requires users to be tech-savvy. Hence, many users choose not to patch the devices in fear of failing to do so properly and damaging the device.
- Many users do not change the default credentials to log into the device. In some cases, these usernames and passwords are hard-coded on the device and cannot be changed.

Mirai, its variants, and many other botnets rely on these vulnerabilities to spread successfully. When an infected IoT device finds an adjacent IoT device, it performs scanning to find the type of the device and then tries to brute-force the username and password using previously known default usernames and passwords for that particular device type, or exploit known vulnerabilities to gain access. Once logged in, the malware spreads to the next device and starts searching for more devices to infect.

### 2.2. Problem Formulation

As the number of IoT devices targeted by attacks increases rapidly, actions must be taken to protect devices connected to the Internet from being exploited and/or used to attack other valuable resources. As explained above, most attacks start with a phase of reconnaissance to scan networks and devices for vulnerabilities.

Many IoT devices remain without adequate security hardening. These devices are accessible with default usernames and passwords and usually generate plain text traffic. This increases the risk generated by reconnaissance attacks, as reconnaissance significantly increases the probability of the attacker gaining control. This makes the risk associated with the attack very high.

This research aims at building a highly accurate, efficient and explainable detection system for reconnaissance attacks based on ensemble machine learning. The detection and prevention of such attacks help significantly limit the spread of malware in IoT devices and help cripple the expansion of botnets. Our research presents an important compensating security control that can address the major risks created by the lack of proper patching of known vulnerabilities in commercial IoT devices. Such lack of patching originates either from the lack of creating such patches by vendors or the lack of user commitment to the patching process. In both scenarios, the proposed system presents a solution to mitigate this significant risk.

## 3. Previous Works

Prior to the fourth industrial revolution (Industry 4.0), research focused on general intrusion detection systems (IDSs). Reconnaissance attacks were considered one of the categories of attacks detected by IDSs. Although this method works some times, it does not provide the required accuracy in detection due to its focus on a much wider range of attacks than reconnaissance alone. Furthermore, reconnaissance attacks can be easily confused with benign activities carried out by legitimate users.

Here, we focus on the detection of reconnaissance attacks in IoT as a method to mitigate malware spread and botnet risks. Our previous work section is divided into two main areas; classical (rule-based) detection and machine-learning-based detection.

### 3.1. Rule-Based Reconnaissance Detection

In 2016, Patel et al. introduced a rule-based IDS based on a a group of efficiently designed detection rules for port scanning [26]. The proposed system utilizes a well-known rule-based intrusion detection system, named SNORT [27], to drop packets that match the port scanning signatures presented in the proposed system. The proposed work was a static rule-based system that causes issues in adapting to the dynamic nature of most of the the recent attacks. Furthermore, the scanning detection rules built are focused on specific features that can easily be manipulated by attackers.

In 2016 also, Sforzin et al. presented an IoT IDS architecture, named RPiDS [28]. The article proposed the use of the Raspberry Pi as an IDS device running SNORT [27], which is a rule-based open source IDS software. Experiments showed that the proposed architecture based on resource-constrained devices, such as the Raspberry Pi, can effectively serve as IDS in a distributed system such as IoT.

Ananin et al. presented, in 2017, a mathematical model built for port scanning detection based on statistical anomalies triggered by port scanning [29]. The proposed mathematical model is capable of detecting port scanning and identifying the source of the scan. The algorithm is based on analyzing the timing of certain types of packets to detect port scanning. Such algorithms can be easy to manipulate by the attacker, by changing the speed of the port scan. Software implementation showed a detection time ranging between 4–28 s, which is proven to be slower than legacy signature-based detection systems.

Other research directions focused on diverting these attacks instead of detecting them, such as [30].

Rana et al. presented, in 2021, a paper discussing the different types of reconnaissance attacks on IoT devices, and explored the dangers of these attacks [31]. The article also discussed some countermeasures that can help reduce the risk of reconnaissance attacks; however, the paper did not present a complete system to counter these attacks.

In general, signature-based intrusion detection systems suffer from a large amount of manual work that must be carried out to update rules and signatures whenever a new attack is identified. In addition, signature-based solutions perform poorly in identifying unknown attacks, compared to machine-learning-based IDSs.

### 3.2. Machine-Learning-Based Detection

The interest in ML-based attack detection has increased in recent years. Whether it is intrusion detection, botnet detection, phishing detection, or other detection types, ML has introduced itself as a viable solution to process large amount of data effectively and efficiently.

In 2018, Viet et al. introduced an anomaly detection system built with deep belief networks (DBNs) [32]. The proposed system combined the use of supervised and unsupervised training to produce a port scanning detection system. Tests were conducted using NSL-KDD [33] and UNSW-NB15 [34] datasets. The proposed DBN system outperformed support vector machine (SVM), naïve Bayes, k-nearest neighbour (k-NN), random forest (RF), decision tree (DT), and multilayer perceptron (MLP) in testing metrics, especially, accuracy. While the tests results were interesting, the datasets used in these tests were captured from computer traffic rather than IoT traffic.

In 2018 also, Meidan et al. presented a network-based anomaly detection method for the IoT called N-BaIoT that extracts snapshots of network behaviour and uses deep autoencoders to detect anomalous network traffic from compromised IoT devices [35]. The features extracted and used in this system were not extracted from a single packet. When a packet arrives, a behavioural snapshot of the hosts and protocols that communicated this packet is taken. The snapshot obtains the context of the packet by computing 115 traffic statistics over several temporal windows to summarize all the traffic that has originated from the same IP in general (source IP). The same set of 23 features are captured from five time windows: the most recent 100 ms, 500 ms, 1.5 s, 10 s, and 1 min. This type of processing to improve online detection is resource-intensive and can cause performance degradation. The proposed systems were tested with the BASHLIE and Mirai IoT botnets and were proven to be capable of detecting multiple stages of malware infection, including scanning.

Anthi et al. presented, in 2018, an IoT-focused IDS named Pulse [36]. The proposed IDS systems were based on machine learning and trained to detect network scanning and simple forms of DoS attacks. The proposed system used a naïve Bayes classifier. The dataset used was created using actual IoT devices. However, it is unclear what the size of the dataset was. The dataset apparently included destination IP addresses, which leads to poor generalization if the model is exposed to new data other than its training dataset. Later, in 2019, the research team published a more detailed paper explaining a more robust approach to creating an IoT IDS system [37]. In this paper, the proposed system was focused on identifying malicious and nonmalicious packets and classifying the type of attack. The article explored the use of nine different types of classifiers, some of which were not successful in detecting a network scan at all. The paper presented better performance compared to the older version.

Huda et al. published, in 2018, a paper proposing an ML-based ensemble system to detect intrusions in SCADA-IoT systems [38]. The paper presents a machine learning model capable of detecting IIoT intrusions with ensemble classifiers. At its best, the proposed system performed with an accuracy of 95.6%. The proposed architecture uses network traffic features and payload features for the detection model instead of signature-based or API-based malware detection technique.

In 2019, Hasan et al. published a paper comparing the performance of multiple machine learning algorithms in attack detection [39]. The paper focused on the detection of seven different classes of attacks, including scanning or reconnaissance attacks. The article presented a very high accuracy of 99.4% achieved using decision tree (DT), RF, and artificial neural networks (ANNs). Although these results look impressive, the paper completely overlooked the problem of dataset imbalance. The dataset has 347,935 normal data and 10,017 anomalous data and contains eight classes that were classified. This means that the benign data class is 97.20%. Using these data without any type of balancing will result in a biased model that will not be able to correctly predict [40]. Therefore, these results cannot be considered valid to compare with.

In 2019, Tzagkarakis et al. presented a botnet detection system based on a sparsity representation framework using a reconstruction error thresholding rule for the detection of malicious traffic at the edge-IoT originating from other compromised devices [41]. The proposed system was trained and evaluated using the N-BaIoT dataset. Experiments showed that the proposed method outperforms autoencoders.

Hafeez et al. presented, in 2020, a lightweight anomaly detection system named IoT-Keeper [42]. The proposed system performs traffic analysis at edge gateways and uses a combination of fuzzy C-means clustering and fuzzy interpolation schemes to analyze network traffic and detect malicious network activity. The proposed technique was tested and provided an accuracy of 0.98 and a false positive rate of 2% to detect malicious network activity.

In 2020, Kim et al. presented a deep-learning-based machine learning system to detect botnet attacks [43]. The proposed system was based on the building of a different machine learning model for each type of IoT device. The dataset used in the experiments was N-BaIoT, and the proposed system achieved an average $F_{1}$ score of 99%.

Again in 2020 [44], Nsabimana et al. proposed a hybrid intrusion detection and prevention system based on hierarchical radial-basis function neural networks, aimed at visualizing, modelling and classifying both known and entirely novel attack instances. Their system addresses the problem of dynamic detection and prevention of unknown (zero-day) attacks, using a mixture of signature-based and unsupervised anomaly-based techniques. The approach integrates unsupervised principal component analysis (PCA) for feature dimensionality reduction.

Sudharsan et al. presented, in 2021, a botnet detection system designed for resource-constrained IoT devices named Edge2Guard [45]. The proposed system is claimed to be a resource-friendly standalone attack detection model. The proposed work was trained and tested using the N-BaIoT dataset, where the 115 features presented in the dataset were reduced to two features only using the principal component analysis. Experiments showed that the proposed system provided an accuracy exceeding 99%. However, the research ignored the fact that principal component analysis is a resource-intensive process that the resource-constrained IoT device would have to perform for every packet received. This would cause severe performance degradation.

In 2021, Alhowaide et al. published a paper also proposing the use of an ensemble classification model to detect intrusions in IoT network traffic [46]. The proposed models showed scores of 0.99, 0.95, 1, and 0.99 $F_{1}$ scores on NSL-KDD, UNSW-NB15, BoTNeTIoT, and BoTIoT datasets, respectively.

In 2022, Alani presented, in [20], a packet-based explainable machine-learning-based detector focused on detecting botnets. Although botnets employ different types of traffic, reconnaissance attacks are an important type of traffic that can originate from botnets, as explained earlier. The proposed system achieved an accuracy exceeding 99% using seven features as well, with the selected features explained using Shapley additive explanations. Other works have also tackled the use of explainable machine learning in attack detection, such as [47,48,49].

In 2023, Alani et al. presented an IoT intrusion detection system with two independent layers operating with packet-based and flow-based features [50]. In this system, the features were extracted at both levels to feed into two independently-trained classifiers to produce a prediction. Although the work did not focus on reconnaissance attacks alone, it is relevant to our proposed system because reconnaissance attacks are included in the dataset used.

### 3.3. Deep Learning-Based Detection

Deep Learning (DL) classifiers have gained tremendous popularity in recent years due to novel training algorithms targeting multilayer architectures and the developments in CPU and GPU processing power, which brought about a noticeable decrease in training cost. The major issue that hinders the adoption of DL solutions in IoT is that most DL systems retain some training capacity in production, and resource-intensive DL training algorithms take a large toll on resource-constrained IoT devices.

Hussain et al. presented, in 2021, a DL-based solution to detect botnet attacks [51]. While a significant percentage of their training set focused on DDoS attacks, botnets included in the dataset utilize reconnaissance attacks intensively to find a vulnerability that can be exploited to gain access to the scanned device. The proposed method relied on deep neural networks and was capable of achieving an $F_{1}$ score of 98.87%.

In 2021, Alani also presented an ML-based detection system aimed at detecting reconnaissance attacks on IoT devices [5]. The proposed system presented an accuracy of 98% using a multilayer perceptron with five hidden layers fed with 12 features. The presented system showed low latency and was capable of producing a prediction in 13.5 μs. While the accuracy is noticeably high, the limited resources available in IoT devices made it computationally challenging to efficiently operate this multilayered solution on IoT devices, even if the perceptron’s number of layers had been kept intentionally limited.

Popoola et al. presented a botnet detection system for IoT edge devices based on federated learning [52]. The proposed system focuses on preserving the privacy of the system users by avoiding the need to collect their data and train a DL classifier centrally. Instead, the global DL model is produced by a central node by synthesizing the parameters of small local learners trained on IoT edge devices. The proposed system was tested using Bot-IoT and N-BaIoT datasets and was able to produce an accuracy of 98.5% and an $F_{1}$ score of 95.56%. Although this direction seems promising, the need to train local models, regardless of how simple they may be, on the devices, as well as the communication overhead of federated learning, are unrealistic for low-end IoT devices.

Qiao et al. presented, in 2021, a dynamic sliding window method that is based on residual subspace projection to examine the impact of concept drift analysis on the performance of cyberattack detection in IoT scenarios [53]. The proposed system does not rely on labels or statistics to identify malicious traffic. Instead, it analyzes the data to detect concept drifts. The proposed system utilizes two types of neural networks: convolutional neural networks (CNNs) and long short-term memory (LSTM) neural networks. CNN achieved an average accuracy of 98.23%, while LSTM achieved 97.06%.

Table 1 shows a comparative summary of the previous works reviewed in contrast to the proposed system.

As shown in the above comparison table, our proposed system is the only one that operates in client–server mode and offsets the processing load of the ML model to a server, away from the resource-constrained IoT devices. Although other systems achieved high accuracy, such as [45,46], they fail to deliver a practical solution that does not overload IoT devices with intensive calculations. Our proposed system was the only solution operating at the packet-level features, rather than the network flow features. This makes it more effective in detecting reconnaissance attacks, as most of these attacks are single-packet attacks such as port scanning, which is commonly carried out using the first packet in the TCP three-way handshake [54]. Network-flow-based systems face latency problems when waiting for the network flow collector to time out and then extract the features and forward them to the machine learning classifier [10].

## 4. Proposed System

**Figure 3:**
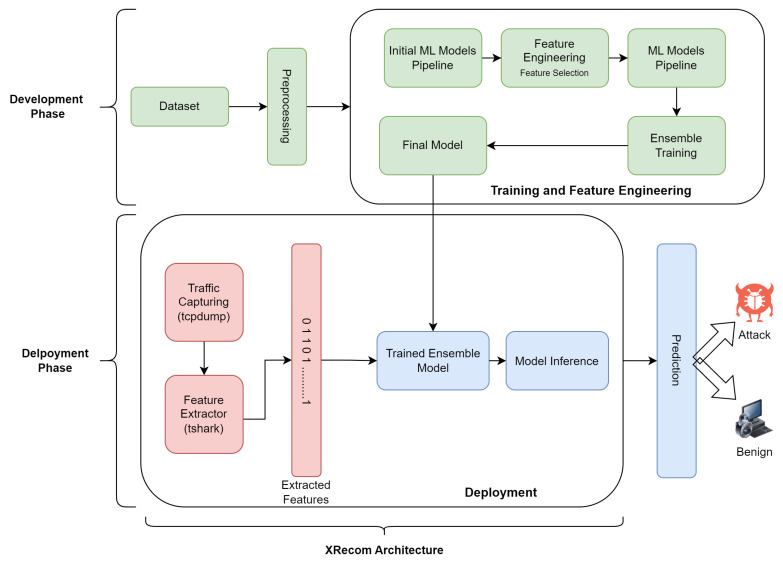
An overview of the proposed system.

An overview of our proposed system is shown in Figure 3. The lifecycle of the system includes two phases; a development phase and a deployment phase.

In the first phase, the ML classifier is created, trained, tested, and validated. Once optimal performance is reached, the trained classifier is stored for later deployment. Further details of model preparation are explained in Section 6.

As shown in Figure 3, the detection process starts with the capture of traffic packets. Then, the captured packets are passed to the feature extractor. Once the features are extracted, they are passed to the pretrained ML model. This model would produce a prediction. If the model inference is “recon”, the packet source is blocked immediately. If the prediction is “benign”, the packet will be allowed to reach the destination IoT device.

It is especially important to underline the rationale behind our choice to resort to ensemble methods for reconnaissance activity detection. The purpose of classical ensemble learning is to improve the generalization capabilities of ML models [55]. The central idea is to employ multiple ML models and combine their predictions based on a consensus algorithm. When individual predictions from different base models are properly combined, the collective decision has, on average, better accuracy than that of any individual model [56]. However, the diversity of the models must be checked to keep the overall size of the ensemble under control. We know that there is no a priori universal recipe to balance diversity and size in ensemble models. Rather, we will take a pragmatic approach and experimentally design an ensemble system whose component models have enough diversity to bind the detector’s dependence on a specific setting or dataset.

## 5. The Dataset

Although many reviewed papers have used the N-BaIoT dataset, we decided against using it. The N-BaIoT dataset includes features already extracted from network flows, while our work is based on extracting features at the packet level rather than at the flow level. Therefore, the dataset selected for this work is the one introduced in [57], namely IoT-ID. The selected dataset was collected in a testbed of NUGU candle and the EZVIZ Wifi camera, which are popular IoT devices, in addition to benign computers and mobile phones. These two IoT devices were infected with the Mirai botnet. The malicious and benign traffic was captured as a group of 42 pcap files in different time instances during the attack and nonattack times. The captured pcap files can be classified as shown in Table 2.

To maintain the focus of our research on reconnaissance attacks, we extracted the packets captures in the host port scanning and port OS scanning categories. Once combined, these pcap files contained 312,561 packets.

The dataset was in the form of raw packet captures. Therefore, the tshark [58] tool was utilized to extract the features from pcap files and output them in comma separated values (CSV) format to simplify the training and testing of the proposed ML model. These features were as follows.

1. frame.len: Layer 2 frame length, measured in bytes.
2. ip.proto: Protocol number, as identified in the IP packet.
3. ip.len: IP packet length.
4. ip.ttl: IP packet Time-To-Live (TTL).
5. ip.flags: The flags field extracted from the IP packet header.
6. ip.hdr_len: The number of bytes in the IP packet header.
7. arp: If the packet is an ARP packet, this feature would hold the specific type of the packet, or it would be empty if it is not ARP.
8. tcp.flags.syn: SYN flag (0 or 1), as extracted from the TCP header.
9. tcp.flags.ack: ACK flag (0 or 1), as extracted from the TCP header.
10. tcp.flags.reset: RESET flag (0 or 1), as extracted from the TCP header.
11. tcp.window_size: Window size, extracted from the TCP header.
12. icmp: An indicator of whether this is an ICMP packet or not.
13. tcp.checksum.status: Indicates the status of the checksum field in the TCP header.
14. tcp.dstport: Destination TCP port numbers.
15. tcp.srcport: Source TCP port number.
16. tcp.flags: Flags field within the TCP header.
17. tcp.len: Length of TCP segment.
18. tcp.time_delta: The time delta between the arrival of the current TCP segment and the previous TCP segment.
19. tcp.urgent_pointer: Urgent pointer in the TCP header.
20. udp.srcport: Source UDP port.
21. udp.dstport: Destination UDP port.

These features were selected based on a thorough statistical study of the differences in packet components between benign and malicious traffic. Data were labelled according to the labelling guidelines provided by the creator of the dataset [57]. In the above list, we carefully removed all host-specific features, such as IP addresses and MAC addresses, to ensure that the trained model can generalize well beyond the training dataset.

The feature extraction phase resulted in the creation of a dataset of 312,051 instances. Each instance holds 21 features extracted from one packet. On thorough examination of the dataset, we made the following observations:

- The features icmp and arp were in text format.
- The features ip.flags and tcp.flags were in hexadecimal representation.
- There were features that had mutually exclusive values. Specifically, all TCP-related features, such as tcp.flags, tcp.checksum.status, and tcp.len, would be missing in instances extracted from UDP packets, and vice versa.
- There is a noticeable imbalance between the malicious class and the benign class, with 286,841 instances of benign and 25,210 instances of malicious packets.

To address the observations listed above, we developed the preprocessing steps shown in Algorithm 1.
**Algorithm 1:** Packet-based dataset preprocessing
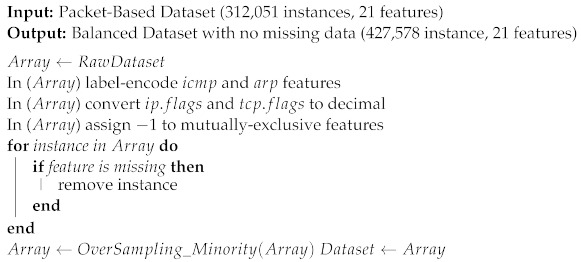


As shown in Algorithm 1, the first step of preprocessing was to perform label-encoding on the features regarding ICMP and ARP. For ICMP packets, the icmp feature value is set to 1, and would be 0 otherwise. For ARP feature, it would be assigned the values 0, 1, 2, and 3, respectively, corresponding to not-ARP, ARP broadcast, ARP request, and ARP response. The second step was to convert the ip.flags and tcp.flags features from hexadecimal to decimal representation. With regard to mutually exclusive features, we chose to assign the missing ones the value −1, as 0 is a valid value for these features. Therefore, all UDP features have a value of −1 in TCP packet instances, and all TCP features have a value of −1 in UDP packet instances. The next step was to remove data instances with missing data features. The last step was to reduce the imbalance between the two classes. We chose to randomly oversample attack packets to the point where the dataset was 66.66% benign and 33.33% malicious. We argue that, in this case, oversampling the minority class is preferable to undersampling the majority one for two reasons. First, our preprocessing phase eliminated all host-specific features, reducing the risk of overfitting linked to undersampling. Second, intuition suggests that benign behaviour has higher variance than malign, and random undersampling of benign behaviour could result in losing information valuable for the model.

As a result of this preprocessing, the resulting dataset consisted of 427,578 instances (285,052,440 benign and 23,720 malicious) without missing data.

## 6. Experimental Setup

### 6.1. Training and Testing Strategy

To achieve the design goals of the proposed system, a training and testing strategy consisting of five phases was devised.

1. The preprocessed dataset is to be randomly divided into a 75% training subset and a 25% testing subset. To maintain class balance, the random split is performed in a stratified manner so that 33.33% of the training subset is “malicious” traffic, and the same percentage of malicious traffic is maintained in the testing subset.
2. A pipeline of five different classifiers is to be built, trained, and tested using the training and testing subsets. The performance of these five classifiers will be used to determine which classifiers deliver better performance.
3. The classifier with the best performance metrics would be used in the next feature selection process. The purpose of this process is to select a lower number of features to improve efficiency and reduce the processing requirements on the IoT device due to its limited resources. The algorithm used in feature selection was RFE based on feature importance. The steps of the algorithm are shown in Algorithm 2.
4. Once the reduced dataset is ready and randomly split, the five classifiers will be trained and tested to ensure that the feature selection process did not significantly impact performance metrics.
5. The results of the previous step are used to select the three best-performing classifiers to use them in constructing a voting-based ensemble to improve the overall performance of the system. The ensemble is then trained and tested with the reduced feature dataset to obtain final system performance metrics.
6. For further validation, the ensemble classifier went through a 10-fold cross-validation process, as shown in Algorithm 3. In a 10-fold cross-validation process, the data are split into 10 subsets randomly. Then, the data go through 10 cycles of training and testing. In each cycle, one subset of the ten is excluded from the training process and used instead for testing. This is repeated ten times until all ten subsets have been used to test one time. Each cycle produces a classifier with specific performance parameters. If these parameters have high variance, then the classifier is diagnosed as suffering from overfitting and does not generalize properly within the dataset. If the variance is low, then the mean values of the performance parameters are reliable results.
7. To further ensure that the classifier generalizes well beyond its training dataset, a second dataset will be used for testing purposes. Performance metrics will be measured for data randomly selected from a second dataset to ensure generalization.
8. As a practical testing step, the trained ensemble classifier will be deployed on an IoT device and an attacking machine will be used to perform reconnaissance attacks to measure performance metrics in a real-life scenario.
9. The final experimentation step is to generate SHAP values for the ensemble classifier to help explain the impact of each feature on the classifier’s prediction.

**Algorithm 2:** Recursive feature elimination using feature importance

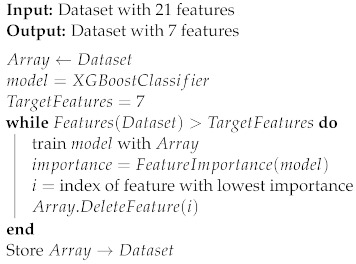



**Algorithm 3: **10-fold cross-validation algorithm

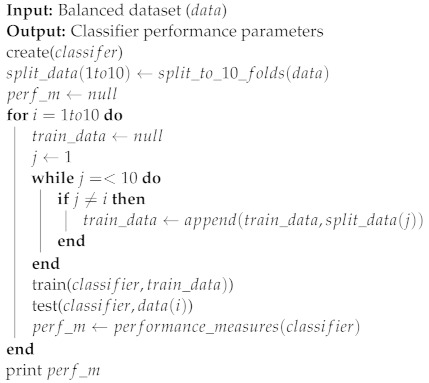



### 6.2. Implementation Environment

Table 3 and Table 4 show the hardware and software specifications of our implementation environment, respectively. The same computer was used to preprocess, train, and test machine learning classifiers.

The practical testing part was conducted on an IoT device named Libre Computer AML-S905X-CC with 4 ARM Cortex-A53 processors operating at 1.512 GHz, and 2 GB of RAM, running Ubuntu v22.04.1, and Python 3.8.5 as well.

### 6.3. Performance Metrics

The basic four performance measures of a binary classifier are:

1. True positive (TP): The number of test instances whose true value and predicted value is 1, divided by the number of test instances whose true value is 1.
2. True negative (TN): The number of test instances whose true value and predicted value is 0, divided by the number of test instances whose true value is 0.
3. False positive (FP): The number of test instances whose true value is 0 and predicted value is 1, divided by the number of test instances whose true value is 0.
4. False negative (FN): The number of test instances whose true value is 1 and predicted value is 0, divided by the number of test instances whose true value is 1.

These four measures, when combined together, generate the confusion matrix. In our research, the following six performance metrics are measured. A detailed description of these metrics can be found in [40].

1. Accuracy
  (1) $$Accuracy=\frac{TP+TN}{TP+TN+FP+FN}$$
2. Precision
  (2) $$Precision=\frac{TP}{TP+FP}$$
3. Recall
  (3) $$Recall=\frac{TP}{TP+FN}$$
4. $F_{1}$ score
  (4) $$F_{1}\;score=2*\frac{\frac{TP}{TP+FN}*\frac{TP}{TP+FP}}{\frac{TP}{TP+FN}+\frac{TP}{TP+FP}}$$
5. Training Time
  The time required to train the classifier using the training subset.
6. Testing Time
  The time required for the trained classifier to process one input instance and produce a prediction.

## 7. Implementation Results

### 7.1. Initial Training and Testing with 21 Features

Our selected feature selection approach, namely, RFE, requires the use of an auxiliary classifier to calculate feature importance. Therefore, we chose five types of initial classifiers to use in our initial training and testing stage. These five classifiers are RF, logistic regression (LR), DT, Gaussian naïve Bayes (GNB), and extended gradient boost (XGB). We selected these five classification algorithms over artificial neural networks (ANNs) because ANNs are known to be more computationally expensive compared to classical ML algorithms [59]. As our research problem is relevant to resources-constrained devices, we did not use ANNs.

Table 5 shows the test results for the five classifiers trained with the 21-feature version of the preprocessed dataset.

As shown in Table 5 the RF, DT, and XGB models delivered better performance metrics compared to LR and GNB. On closer examination, we noticed that XGB slightly outperformed RF and DT. Hence, we choose the XGB classifier to be used in the feature selection process.

### 7.2. Feature Selection Using RFE

**Figure 4:**
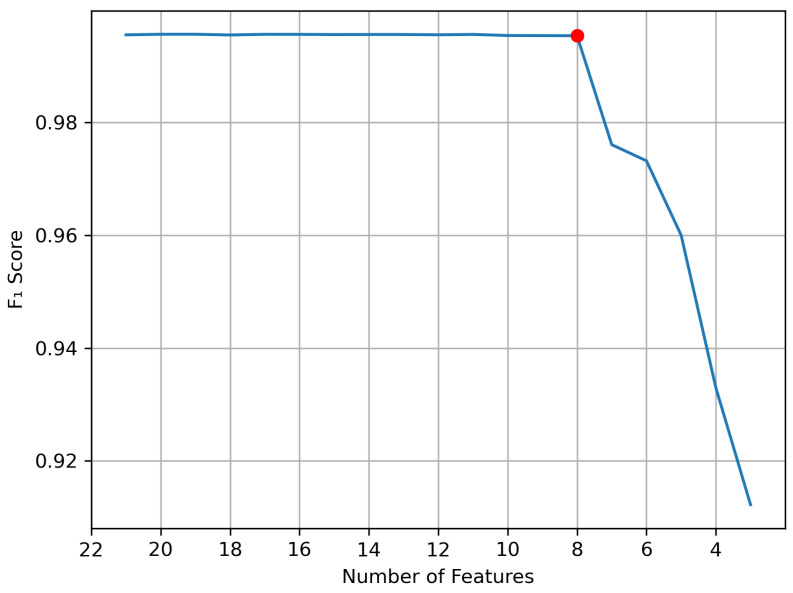
Change in $F_{1}$ score with feature reduction.

The RFE feature selection algorithm is shown in Algorithm 2. As shown in the algorithm, the XGB classifier is trained and tested to calculate its $F_{1}$ score. Then, the feature importance is calculated. In decision-tree-based models (such as DT, RF, and XGB classifiers), features’ importance scores are calculated based on the reduction they bring to the score used to select split points, usually entropy. Once all features’ importance have been calculated, the feature with the lowest measured importance is removed from the dataset, and another cycle of training and testing starts. While these cycles are occurring, the $F_{1}$ score of the classifier is monitored to identify the specific number of features beyond which the performance degrades significantly. We chose this method of feature selection over other methods well documented in the literature such as principal component analysis (PCA) and linear discriminant analysis (LDA) due to the high processing cost of these algorithms. RFE would not only reduce the number of features sent to the classifier but also reduce the number of features that need to be captured and extracted during the data acquisition phase in production. Figure 4 shows the change in $F_{1}$ score with the reduction in features.

As shown in Figure 4, the final number of features is eight, as a lower number of features caused a significant drop in the $F_{1}$ score. Our feature selection process resulted in producing a much smaller version of the dataset with eight features only.

To measure the impact of the feature selection process on the classifiers’ performance, we retrained and tested the five classification algorithms selected earlier using the eight-feature version of the dataset. Table 6 shows the performance metrics resulting from this testing phase.

According to Table 6, the performance of RF, DT, and XGB was not significantly impacted by feature reduction. On the other hand, LR performance was significantly poorer. On the basis of these results, we selected RF, DT, and XGB to build our ensemble classifier.

### 7.3. Ensemble Training and Testing with 8 Features

The next step was to build an ensemble classifier using the three classifier types identified earlier. The type of the ensemble we chose is the voting ensemble, which produces a prediction that is the average of the outputs of the component models. The type of voting chosen for our model is “hard” voting. In hard voting, the predicted label is chosen by picking the label that was predicted by most of the ensemble classifiers.

**Figure 5:**
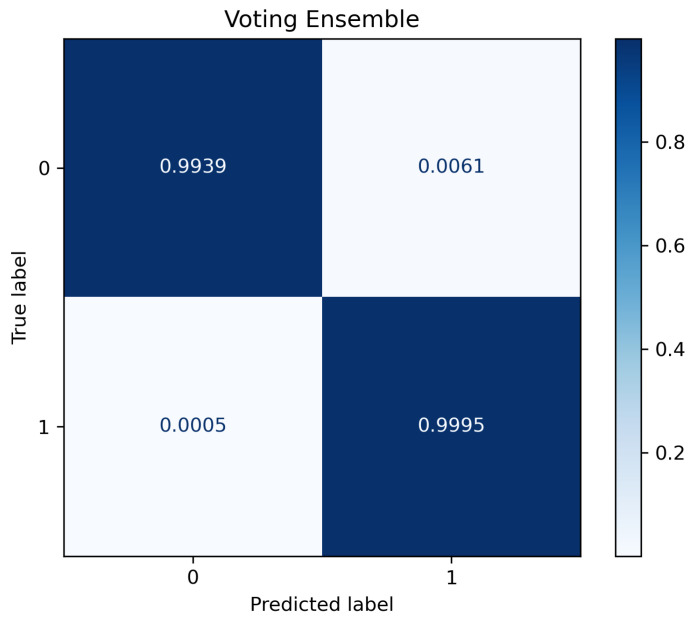
Confusion matrix plot for ensemble model.

Table 7 shows the classification matrix for testing the ensemble classifier after training. As the table shows, the ensemble classifier outperformed the individual classifiers tested in the previous step. This is shown in the slightly higher $F_{1}$ score of 0.995235, as well as in the other performance metrics. The measured training time for the ensemble was 17.643 s, while the testing time was 14.2 μs. Figure 5 shows the confusion matrix plot for the ensemble classifier. As shown in the figure, the classifier showed a very low FP rate of 0. 61% and an FN rate of 0.05%.

### 7.4. 10-Fold Cross-Validation of Ensemble Classifier

In this validation step, the proposed system was subjected to a 10-fold cross-validation procedure, as shown in Algorithm 3. Table 8 shows the results of this process.

As shown in the table, the performance metrics measured were consistent with the previous testing phase. All performance metrics also showed a very small standard deviation. This means that the results obtained are robust and that the trained model can generalize well beyond its training dataset.

### 7.5. Testing with a Second Dataset

**Figure 6:**
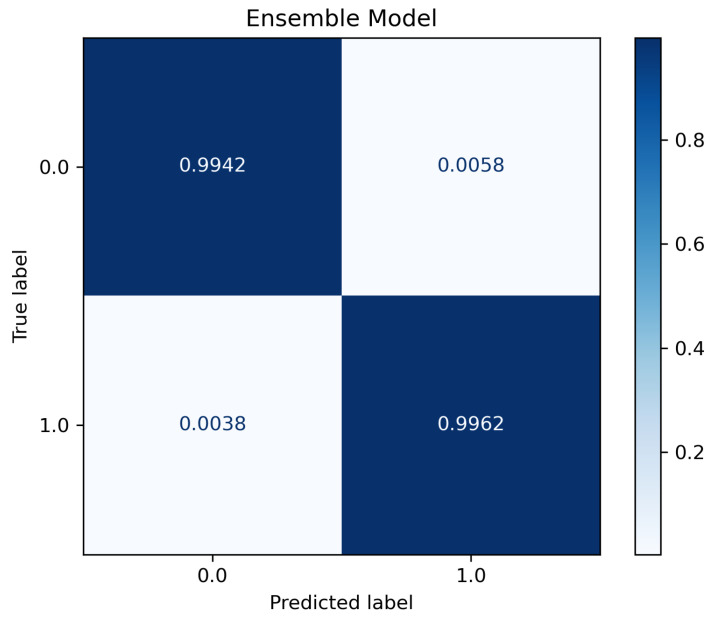
Confusion matrix plot for ensemble model using TON_IoT dataset.

To further validate the results obtained and verify the dataset independence of our results, we extracted the scanning packets from the TON_IoT dataset [60] to test our trained ensemble classifier. The TON_IoT dataset was collected from IoT and IIoT devices infected with different types of malware. We randomly selected 10,000 reconnaissance packets from the dataset and extracted the same eight features that were selected earlier. Table 9 shows the testing results using 10,000 samples of TON_IoT dataset, and Figure 6 shows the confusion matrix plot for this testing phase.

As shown in the table and figure, the performance metrics are highly aligned with the results obtained during the previous two steps. This proves that our ensemble classifier generalizes well beyond its training dataset.

### 7.6. Practical Deployment and Testing

**Figure 7:**
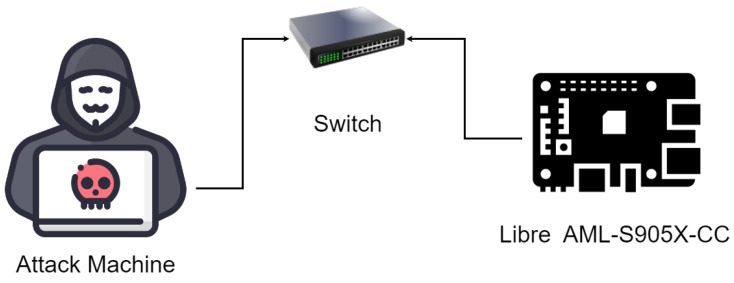
Practical deployment topology.

To measure the performance of the proposed system in production, we tested it in a real-world environment. The environment topology used in the test is shown in Figure 7. In this testing topology, the target IoT device, Libre Computer AML-S905X-CC, was used. The target machine ran Ubuntu v22.04.1, with a Python environment similar to the one used in training and testing. In this client, the trained ensemble classifier was deployed along with two additional tools to capture packets and extract features. For packet capture, tcpdump [61], was used, and for feature extraction, tshark was used. A small Python program was written to facilitate the interfacing between the packet capture tool, the feature extraction tool, and the trained classifier. The operation at the target is summarized in Algorithm 4.
**Algorithm 4: **Target system operation
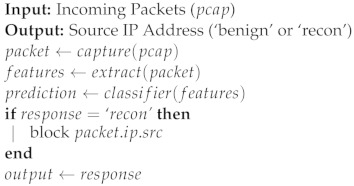


    On the attacker side, Kali Linux was installed and the nmap tool was used to perform scanning attacks. A script was written to run 1000 attacks in a period of 24 h. The script would change the IP address of the attack machine after each attack to avoid being blocked. The target system succeeded in detecting 100% of the scanning attempts and blocking the source IP address. The average detection time was 27.2 μs, including the packet capture time and the extraction time of features.

## 8. Model Explanation

We use Shapley additive explanation (SHAP) to provide the explainability of our trained model. The algorithm behind SHAP was presented in [62], in 2017. Its strength lies in being a model-agnostic method used in explaining various types of machine learning models. The algorithm is based on Shapley values in game theory. The algorithm calculates the impact of each feature by examining the performance difference of the model with and without the selected features. This provides an understanding of the particular contribution the feature makes to the prediction produced by the classifier. The explainer used in our experiment was TreeExplainer, which is capable of calculating the explainability for all of the classifiers used in building our ensemble. The method by which the SHAP values were calculated for the ensemble is to find the SHAP values of each of the contributing classifiers and averaging these values to generate the SHAP values for the whole ensemble [63].

**Figure 8:**
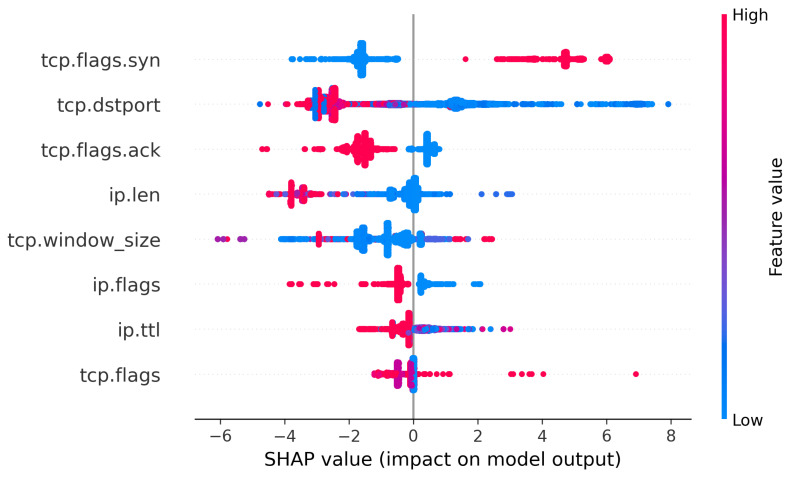
SHAP summary plot for ensemble model.

Figure 8 shows the SHAP values summary plot of the eight features selected in our proposed system. These features are ordered in descending order from the feature with the highest impact on the decision at the top to the lowest impact at the bottom of the figure.

The dots appearing on the left side of the summary plot are those that result in reduction in the prediction value to bring it closer to the “benign” class. Dots on the right side of the figure indicate causing a higher prediction value, pushing the prediction closer to “recon” class. When in red, these dots carry a high value of the feature, while when in blue they represent a low value.

As shown in the figure, the feature with the highest impact is tcp.flags.syn. This feature has the value of 1 if the SYN flag is set in the TCP segment, and the value of 0 if this flag is not set. The figure shows that an SYN flag value of 1 pushes the prediction closer to “recon”. This is consistent with the fact that most reconnaissance packets are sent with this flag set to start the TCP three-way handshake. This is also confirmed by examining the third feature in the figure, tcp.flags.ack. In most TCP reconnaissance packets, the value of the ACK flag is set to 0 when the attacker starts a TCP session. Therefore, when tcp.flags.ack is not set, the prediction is pushed in the “benign” direction. It is also worth mentioning that scanning with both ACK and SYN set is popular. However, this form of scanning requires high-privilege access on the attacking device. While that may be available to a live attacker, it might not be available for scanning scripts that run from infected IoT devices such as botnets.

The second feature shown in the figure is tcp.dstport. This feature holds the destination port number in the TCP segment. The figure shows that the lower values of the destination port number push the prediction to “recon”. This is consistent with the fact that most port scanning attacks focus on port numbers of well-known ports, ranging from 1 to 1024. This is the range where most targeted protocols operate, such as HTTP on port 80, SSH on port 22, and FTP on ports 20 and 21. While there are some blue dots on the left side of the figure, the prevailing amount of scans are performed on the lower port numbers.

The fourth feature shown in the figure is ip.len, which holds the number of bytes within an IP packet. The figure shows that higher values of this feature push the prediction decision to “benign”. This is consistent with the fact that most reconnaissance attacks are conducted using small-sized probing packets that do not hold significant amounts of data.

The next feature in the figure is tcp.windows_size, which holds the windows size field extracted from the TCP header. The figure shows that lower values of the windows size are more coherent with benign traffic. In most scenarios, the attack is performed within the local networks, which is usually associated with larger windows size due to lower error rates [54].

Another feature, ip.flags, is extracted from the flags field within IP packets. The figure indicates that the lower value of this field is associated with reconnaissance packets, while the higher values are associated with benign traffic. The flags field is composed of three bits; a zero bit, a do not fragment bit, and a fragmented bit. The possible values of these bits are 000, 010, and 011. Higher values indicate that the packet can be fragmented, and might either be the last packet in a fragmented group or just another packet within a fragmented group. This also indicates that a packet that is not fragmented is more likely to be an attack packet. Reconnaissance packets are usually individual probing packets and do not require fragmentation due to their relatively smaller payload. This is in agreement with our previous explanation of the ip.len feature.

Higher values of the ip.ttl feature suggest benign traffic, as shown in the SHAP summary figure. This is due to the fact that most of the scanning tools used by attackers, such as nmap, use a small default TTL value. However, this is not an explicit feature for attack packets. Many benign packets have low TTL values, as shown in the figure.

The feature with the least impact on the prediction was tcp.flags which combines the values of the eight TCP header flags: ACK, FIN, URG, PSH, RST, ECE, CWR, and NS. These flags have individual bit values, which are combined to create a 2 bytes flags field. The figure does not show a clear distinction of values of the flags field and how they impact the prediction. However, a general observation is that higher values of the flags field, which are associated with the ACK flag being set, push the prediction to the benign side. This is consistent with our previous explanation of the tcp.flags.ack feature.

## 9. Discussion

The proposed system was extensively tested at multiple development and deployment levels to ensure high accuracy, efficient performance, and generalization beyond the training dataset. For generalization purposes, multiple steps were taken. Host-relevant features, such as source and destination addresses, were removed in the preprocessing stage to ensure that the classifier does not rely on these host-specific features to make the prediction. Furthermore, testing was performed using not only the testing subset of the original dataset, but also realistic Nmap scans and data from another well-known dataset were used. In all three scenarios, the proposed system performed superbly.

For the sake of comparison, we were unable, to the best of our knowledge, to find a similar system that was deployed on realistic devices to compare with. Therefore, our comparison to previous work will be limited to comparing the performance of the ML classifier. Table 10 shows the comparison of our proposed classifier with other previous work based on ML.

The comparison table shows the dataset used, along with the number of features, and identifies the type of feature extraction as packet-based or flow-based. In addition, the comparison shows the ML algorithm used along with the performance metrics, and whether the classifier was explainable or not.

From Table 10, we can see that our proposed classifier performs with slightly higher accuracy than [38,65]. Although [32] reports slightly higher accuracy, the classifier used in that research is based on neural networks, while our classifier utilizes an ensemble of classical ML algorithms, which are known to be less resource-hungry and faster in categorical data.

Previous works [66,67,68] were selected for comparison because they utilize the TON_IoT dataset that was used in one of our testing stages. As Table 10 shows, our proposed system, when tested with the TON_IoT dataset, outperformed previous work by a large margin in terms of accuracy.

The only previous ML-based work that we found that is focused specifically on reconnaissance attacks is [32]. However, the dataset used (UNSW-NB15) does not contain IoT or IIoT traffic, but general IDS traffic. In comparison, the dataset we used included only IoT/IIoT traffic. The other previous works listed proposed general IDS solutions without specific focus on reconnaissance attacks; however, their datasets included some reconnaissance data.

Upon examining the previous works listed, we found that most of the works did not undergo extensive testing to prove their ability to generalize in a way similar to that of our proposed system. We also found that none of the proposed systems were tested on actual IoT devices. Hence, we cannot compare with them in terms of generalization.

The work presented in [32,38,66,68] utilized different types of ANNs. Neural networks are more resource-intensive compared to classical ML algorithms [40]. On the other hand, [65] utilized B-stacking where three base learners (k-neighbours, RF, and XGBoost) were used together with XGBoost as a meta-classifier. Our proposed system uses a simpler voting-based ensemble classification that uses only eight features. Another downside of [65] is that it was trained and validated using a general IDS dataset, rather than an IoT one.

Another advantage of the proposed system in comparison to other systems is that it operates at the packet level. This makes it more efficient in detecting single-packet reconnaissance attacks such as port and network scanning.

As the table shows, none of the previous work reviewed utilized any type of explainability techniques. Such techniques would increase trust in our proposed model and ensure that prediction decisions do not originate from a “black-box”.

## 10. Conclusions and Future Work

In this paper, we presented an efficient and explainable system to detect reconnaissance attacks in IoT environments supported by machine learning. The proposed system was tested in several stages and performed with an average accuracy of 99.57%. The machine learning classifier was deployed on an IoT device that was subjected to live reconnaissance attacks. Once a “recon” prediction is generated, the IoT device blocks the sender and stops the attack.

The proposed system was intensively tested using different scenarios to ensure that it can properly generalize beyond the training dataset, within dataset testing, a second dataset testing, and actual Nmap scans. These tests returned an accuracy of 99.57%, 99.49%, and 100%, respectively.

The work presented can be further extended by exploring other ML algorithms as base models depending on the balance between diversity and size suitable for a specific application domain. Another direction is to measure the complexity of the proposed system and explore ways to reduce this complexity in resources-constrained devices. A closely related future direction is to examine the deployment of the proposed system in different contexts such as smart cities, industrial IoT, and healthcare settings. Another interesting research direction is to explore the impact of data drift over time in various datasets.

## Figures and Tables

**Table 1:** Comparative summary of selected previous Works.

Paper	Algorithm	Dataset	Features	Traffic	Accuracy	Detection	Lightweight	Explainable
[38]	Ensemble DBN	SCADA2014		Flow	95.6%	IIoT Intrusion		
[52]	fed-DNN	Bot-IoT and N-BaIoT	43	Flow	98.5%	Botnet		
[39]	RF	DS2OS	13	Flow	99.4%	Intrusion		
[32]	DBN	UNSW-NB15 and NSL-KDD	49 and 41	Flow	99.45%	Recon		
[35]	Deep autoencoder and IsolationForest	N-BaIoT	115	Flow	75%	Intrusion		
[36]	Naive-Bayes	[36]	5	Flow	97.7%	Scanning		
[51]	DNN	CICIDS-19, CICIDS-17, and Bot-IoT	15	Flow	98.7%	Botnet	✓	
[45]	RF and DT	N-BaIoT	2 (PCA)	Flow	99%	Botnet		
[46]	Ensemble	BoTNetIoT	23	Flow	99%	Intrusion		
[44]	RBF	Bot-IoT	7 (PCA)	Flow	98.7%	Botnet	✓	
[50]	XGB	IoT-ID	9 and 5	Both	99.15%	Intrusion		
Proposed		IoT-ID	7	Packet	99.51%	Recon	✓	✓
		TON_IoT	7	Packet	99.51%	Recon	✓	✓

**Table 2:** Categories of packets captured in the dataset.

Packet Category	Number of Files
Benign	1
DoS Syn-Flood	6
MITM ARP Spoofing	6
Host Port Scanning	6
Port OS Scanning	6
Mirai Acknowledgement Flood	4
Mirai Host Bruteforce	5
Mirai HTTP Flood	4
Mirai UDP Flood	4

**Table 3:** Hardware specifications of the implementation environment.

Processor	AMD Ryzen 5 3600
Clock Speed	4.2 GHz
RAM	128 GB
GPU	NVidia GeForce RTX 3060Ti

**Table 4:** Software specification of the implementation environment.

Operating System	Windows 10 Pro
Python	3.8.5
Sci-Kit Learn	1.0.1
tshark	3.4.4
VMWare Workstation Pro	16.2.0

**Table 5:** Initial testing performance metrics.

Model	Accuracy	Precision	Recall	$F_{1}$ Score	Train Time (s)	Test Time (μs)
RF	0.985925	0.984232	0.986651	0.985427	11.955842	6.237922
LR	0.719889	0.721772	0.749461	0.712425	7.571759	0.042521
DT	0.985386	0.983868	0.985790	0.984820	0.440740	0.112022
GNB	0.677919	0.667538	0.687639	0.664492	0.099769	0.332227
XGB	0.986301	0.984512	0.987225	0.985850	4.664476	0.450452

**Table 6:** Testing results after feature selection.

Model	Accuracy	Precision	Recall	$F_{1}$ Score	Train Time (s)	Test Time (μs)
RF	0.985882	0.983992	0.986811	0.985381	11.887003	8.114719
LR	0.666863	0.833398	0.500298	0.400642	1.592001	0.014165
DT	0.985521	0.983694	0.98629	0.984974	0.264004	0.077943
GNB	0.666898	0.6685	0.500521	0.401407	0.066998	0.155914
XGB	0.986017	0.984096	0.987013	0.985532	2.965999	0.382704

**Table 7:** Ensemble classification matrix.

Class	Precision	Recall	$F_{1}$ Score
0	0.999760	0.993895	0.996819
1	0.987852	0.999520	0.993652
Macro Avg	0.993806	0.996708	0.995235
Weighted Avg	0.995808	0.995762	0.995768
Accuracy	0.995762		

**Table 8:** Results of 10-fold cross-validation using ensemble model.

Fold	Accuracy	Precision	Recall	$F_{1}$ Score
1	0.995954	0.987884	1.000000	0.993905
2	0.995743	0.987730	0.999649	0.993654
3	0.996001	0.988594	0.999508	0.994021
4	0.996024	0.988517	0.999648	0.994051
5	0.996071	0.988838	0.999654	0.994216
6	0.996585	0.989968	0.999787	0.994854
7	0.996445	0.989677	0.999790	0.994708
8	0.995814	0.987842	0.999861	0.993815
9	0.995603	0.987368	0.999651	0.993471
10	0.996211	0.988785	0.999930	0.994326
Mean	0.996045	0.988520	0.999748	0.994102
St Dev	0.000288	0.000802	0.000144	0.000415

**Table 9:** Ensemble classification matrix for TON_IoT samples.

Class	Precision	Recall	$F_{1}$ Score
0	0.9981	0.9942	0.9962
1	0.9885	0.9962	0.9924
Macro Avg	0.9933	0.9952	0.9943
Weighted Avg	0.9949	0.9949	0.9949
Accuracy	0.9949		

**Table 10:** Comparison of results with previous works.

Paper	Dataset	Features	Traffic	Algorithm	Accuracy	FP	FN	Explainable
[32]	UNSW-NB15	49	Flow	DBN	99.64%	2.76%	-	×
[38]	[64]	-	Flow	Ensemble DBN	95.60%	8.4%	4.8%	×
[65]	CICIDS2017	28	Flow	B-Stacking	99.11%	0.92%	0.89%	×
[66]	TON_IoT	44	Flow	Graph-NN	86.68%	-	-	×
[67]	TON_IoT	44	Flow	Voting	87.71%	-	-	×
[68]	NF-TON_IoT	44	Flow	DNN	83%	-	-	×
Proposed	IoT-ID [57]	8	Packet	Ensemble	99.57%	0.61%	0.05%	✓
	TON_IoT	8	Packet	Ensemble	99.51%	0.7%	0.05%	✓

## Data Availability

Data used in this study is available upon request.

## References

[1] Gubbi J., Buyya R., Marusic S., Palaniswami M. (2013). Internet of Things (IoT): A vision, architectural elements, and future directions. Future Gener. Comput. Syst..

[2] (2021). 2020’s Internet of Things Statistics, Facts & Predictions. https://review42.com/resources/internet-of-things-stats.

[3] (2021). Android|The Platform Pushing What’s Possible. https://www.android.com/intl/en_ca.

[4] The Raspberry Pi Foundation (2021). Operating System Images—Raspberry Pi. Raspberry Pi..

[5] Alani M.M. (2022). Detection of Reconnaissance Attacks on IoT Devices Using Deep Neural Networks. Advances in Nature-Inspired Cyber Security and Resilience.

[6] (2022). IoT Devices Installed Base Worldwide 2015–2025|Statista. https://www.statista.com/statistics/471264/iot-number-of-connected-devices-worldwide.

[7] (2021). OT/IoT Security Report February 2021|Nozomi Networks. https://www.nozominetworks.com/landing/ot-iot-security-report-february-2021/.

[8] Uma M., Padmavathi G. (2013). A Survey on Various Cyber Attacks and their Classification. IJ Netw. Secur..

[9] Yadav T., Rao A.M. Technical aspects of cyber kill chain. Proceedings of the International Symposium on Security in Computing and Communication.

[10] Alani M.M. (2023). An explainable efficient flow-based Industrial IoT intrusion detection system. Comput. Electr. Eng..

[11] (2021). Nmap. https://nmap.org.

[12] (2021). Shodan. https://www.shodan.io.

[13] (2021). Home—Censys. https://censys.io.

[14] (2021). Drupal—Open Source CMS. https://www.drupal.org.

[15] (2021). CVE—CVE. https://cve.mitre.org.

[16] (2021). CVE—CVE-2014-3704. https://cve.mitre.org/cgi-bin/cvename.cgi?name=CVE-2014-3704.

[17] (2021). Metasploit|Penetration Testing Software, Pen Testing Security|Metasploit. https://www.metasploit.com.

[18] (2021). OT/IoT Security Report: Rising IoT Botnets and Shifting Ransomware Escalate Enterprise Risk. https://www.nozominetworks.com/blog/what-it-needs-to-know-about-ot-io-security-threats-in-2020.

[19] Silva S.S., Silva R.M., Pinto R.C., Salles R.M. (2013). Botnets: A survey. Comput. Netw..

[20] Alani M.M. (2022). BotStop: Packet-based efficient and explainable IoT botnet detection using machine learning. Comput. Commun..

[21] Author G. (2020). Inside the infamous Mirai IoT Botnet: A Retrospective Analysis. Cloudflare Blog. https://blog.cloudflare.com/inside-mirai-the-infamous-iot-botnet-a-retrospective-analysis.

[22] O’Donnell L. (2021). Latest Mirai Variant Targets SonicWall, D-Link and IoT Devices. Threatpost. https://threatpost.com/mirai-variant-sonicwall-d-link-iot/164811.

[23] Montalbano E. (2020). New Mirai Variant ‘Mukashi’ Targets Zyxel NAS Devices. Threatpost. https://threatpost.com/new-mirai-variant-mukashi-targets-zyxel-nas-devices/153982.

[24] (2021). Mirai Variant Targeting New IoT Vulnerabilities, Network Security Devices. https://unit42.paloaltonetworks.com/mirai-variant-iot-vulnerabilities.

[25] Kolias C., Kambourakis G., Stavrou A., Voas J. (2017). DDoS in the IoT: Mirai and other botnets. Computer.

[26] Patel S.K., Sonker A. (2016). Rule-Based Network Intrusion Detection System for Port Scanning with Efficient Port Scan Detection Rules Using Snort. Int. J. Future Gener. Commun. Netw..

[27] (2021). Snort—Network Intrusion Detection & Prevention System. https://www.snort.org.

[28] Sforzin A., Mármol F.G., Conti M., Bohli J.M. Rpids: Raspberry pi ids—A fruitful intrusion detection system for iot. Proceedings of the 2016 International IEEE Conferences on Ubiquitous Intelligence & Computing, Advanced and Trusted Computing, Scalable Computing and Communications, Cloud and Big Data Computing, Internet of People, and Smart World Congress (UIC/ATC/ScalCom/CBDCom/IoP/SmartWorld).

[29] Ananin E.V., Nikishova A.V., Kozhevnikova I.S. Port scanning detection based on anomalies. Proceedings of the 2017 Dynamics of Systems, Mechanisms and Machines (Dynamics).

[30] Achleitner S., La Porta T.F., McDaniel P., Sugrim S., Krishnamurthy S.V., Chadha R. (2017). Deceiving network reconnaissance using SDN-based virtual topologies. IEEE Trans. Netw. Serv. Manag..

[31] Rana S., Garg U., Gupta N., Das A.K., Nayak J., Naik B., Dutta S., Pelusi D. (2022). Reconnaissance Attacks: A First Step to Hack IoT Devices and Cyber Crime. Computational Intelligence in Pattern Recognition.

[32] Viet H.N., Van Q.N., Trang L.L.T., Nathan S. Using Deep Learning Model for Network Scanning Detection. Proceedings of the 4th International Conference on Frontiers of Educational Technologies—ICFET’18.

[33] (2020). NSL-KDD|Datasets|Research|Canadian Institute for Cybersecurity|UNB. https://www.unb.ca/cic/datasets/nsl.html.

[34] Moustafa N., Slay J. UNSW-NB15: A comprehensive data set for network intrusion detection systems (UNSW-NB15 network data set). Proceedings of the 2015 Military Communications and Information Systems Conference (MilCIS).

[35] Meidan Y., Bohadana M., Mathov Y., Mirsky Y., Shabtai A., Breitenbacher D., Elovici Y. (2018). N-baiot—Network-based detection of iot botnet attacks using deep autoencoders. IEEE Pervasive Comput..

[36] Anthi E., Williams L., Burnap P. Pulse: An adaptive intrusion detection for the internet of things. Proceedings of the Living in the Internet of Things: Cybersecurity of the IoT 2018.

[37] Anthi E., Williams L., Slowinska M., Theodorakopoulos G., Burnap P. (2019). A Supervised Intrusion Detection System for Smart Home IoT Devices. IEEE Internet Things J..

[38] Huda S., Yearwood J., Hassan M.M., Almogren A. (2018). Securing the operations in SCADA-IoT platform based industrial control system using ensemble of deep belief networks. Appl. Soft Comput..

[39] Hasan M., Islam M.M., Zarif M.I.I., Hashem M. (2019). Attack and anomaly detection in IoT sensors in IoT sites using machine learning approaches. Internet Things.

[40] Géron A. (2019). Hands-On Machine Learning with Scikit-Learn, Keras, and TensorFlow: Concepts, Tools, and Techniques to Build Intelligent Systems.

[41] Tzagkarakis C., Petroulakis N., Ioannidis S. Botnet attack detection at the IoT edge based on sparse representation. Proceedings of the 2019 Global IoT Summit (GIoTS).

[42] Hafeez I., Antikainen M., Ding A.Y., Tarkoma S. (2020). IoT-KEEPER: Detecting malicious IoT network activity using online traffic analysis at the edge. IEEE Trans. Netw. Serv. Manag..

[43] Kim J., Shim M., Hong S., Shin Y., Choi E. (2020). Intelligent detection of iot botnets using machine learning and deep learning. Appl. Sci..

[44] Nsabimana T., Hounsou J.T., Damiani E., Houngbo P., Frati F. (2022). Hybrid Intrusion Detection and Prevention Systems Using Hierarchical Radial Basis Function Neural Networks. SSRN Electronic J..

[45] Sudharsan B., Sundaram D., Patel P., Breslin J.G., Ali M.I. Edge2guard: Botnet attacks detecting offline models for resource-constrained iot devices. Proceedings of the 2021 IEEE International Conference on Pervasive Computing and Communications Workshops and other Affiliated Events (PerCom Workshops).

[46] Alhowaide A., Alsmadi I., Tang J. (2021). Ensemble detection model for IoT IDS. Internet Things.

[47] Alani M.M., Miri A. (2022). Towards an Explainable Universal Feature Set for IoT Intrusion Detection. Sensors.

[48] Abou El Houda Z., Brik B., Khoukhi L. (2022). “Why Should I Trust Your IDS?”: An Explainable Deep Learning Framework for Intrusion Detection Systems in Internet of Things Networks. IEEE Open J. Commun. Soc..

[49] Oseni A., Moustafa N., Creech G., Sohrabi N., Strelzoff A., Tari Z., Linkov I. (2022). An Explainable Deep Learning Framework for Resilient Intrusion Detection in IoT-Enabled Transportation Networks. IEEE Trans. Intell. Transp. Syst..

[50] Alani M.M., Awad A.I. (2022). An Intelligent Two-Layer Intrusion Detection System for the Internet of Things. IEEE Trans. Ind. Inform..

[51] Hussain F., Abbas S.G., Pires I.M., Tanveer S., Fayyaz U.U., Garcia N.M., Shah G.A., Shahzad F. (2021). A Two-Fold Machine Learning Approach to Prevent and Detect IoT Botnet Attacks. IEEE Access.

[52] Popoola S.I., Ande R., Adebisi B., Gui G., Hammoudeh M., Jogunola O. (2021). Federated deep learning for zero-day botnet attack detection in IoT-edge devices. IEEE Internet Things J..

[53] Qiao H., Novikov B., Blech J.O. (2021). Concept Drift Analysis by Dynamic Residual Projection for Effectively Detecting Botnet Cyber-Attacks in IoT Scenarios. IEEE Trans. Ind. Inform..

[54] Alani M.M. (2014). Tcp/ip model. Guide to OSI and TCP/IP Models.

[55] Sagi O., Rokach L. (2018). Ensemble learning: A survey. Wired Data Min. Knowl. Discov..

[56] Mauri L., Apolloni B., Damiani E. (2023). Robust ML Model Ensembles via Risk-driven Anti-clustering of Training Data. Inf. Sci..

[57] Kang H., Ahn D.H., Lee G.M., Yoo J.D., Park K.H., Kim H.K. (2019). IoT network intrusion dataset. IEEE Dataport.

[58] (2021). tshark—The Wireshark Network Analyzer 3.4.5. https://www.wireshark.org/docs/man-pages/tshark.html.

[59] Raschka S., Liu Y.H., Mirjalili V., Dzhulgakov D. (2022). Machine Learning with PyTorch and Scikit-Learn: Develop Machine Learning and Deep Learning Models with Python.

[60] Moustafa N. New Generations of Internet of Things Datasets for Cybersecurity Applications based Machine Learning: TON_IoT Datasets. Proceedings of the eResearch Australasia Conference.

[61] (2021). TCPDUMP/LIBPCAP Public Repository. https://www.tcpdump.org.

[62] Lundberg S.M., Lee S.I. A unified approach to interpreting model predictions. Proceedings of the Annual Conference on Neural Information Processing Systems 2017.

[63] Kamath U., Liu J. (2021). Explainable Artificial Intelligence: An Introduction to Interpretable Machine Learning.

[64] Morris T., Gao W. Industrial control system traffic data sets for intrusion detection research. Proceedings of the International Conference on Critical Infrastructure Protection.

[65] Roy S., Li J., Choi B.J., Bai Y. (2022). A lightweight supervised intrusion detection mechanism for IoT networks. Future Gener. Comput. Syst..

[66] Lo W.W., Layeghy S., Sarhan M., Gallagher M., Portmann M. E-GraphSAGE: A Graph Neural Network based Intrusion Detection System for IoT. Proceedings of the NOMS 2022—2022 IEEE/IFIP Network Operations and Management Symposium.

[67] Khan M.A., Khan Khattk M.A., Latif S., Shah A.A., Ur Rehman M., Boulila W., Driss M., Ahmad J. (2022). Voting classifier-based intrusion detection for iot networks. Advances on Smart and Soft Computing.

[68] Awotunde J.B., Abiodun K.M., Adeniyi E.A., Folorunso S.O., Jimoh R.G. A deep learning-based intrusion detection technique for a secured IoMT system. Proceedings of the International Conference on Informatics and Intelligent Applications.

